# Dissecting the Functional Role of Key Residues in Triheme Cytochrome PpcA: A Path to Rational Design of *G. sulfurreducens* Strains with Enhanced Electron Transfer Capabilities

**DOI:** 10.1371/journal.pone.0105566

**Published:** 2014-08-25

**Authors:** Leonor Morgado, Sílvia Lourenço, Yuri Y. Londer, Marianne Schiffer, P. Raj Pokkuluri, Carlos A. Salgueiro

**Affiliations:** 1 Requimte, CQFB, Departamento de Química da Faculdade de Ciências e Tecnologia da Universidade Nova de Lisboa (FCT/UNL), Caparica, Portugal; 2 Biosciences Division, Argonne National Laboratory, Argonne, Illinois, United States of America; Cardiff University, United Kingdom

## Abstract

PpcA is the most abundant member of a family of five triheme cytochromes *c*_7_ in the bacterium *Geobacter sulfurreducens* (*Gs*) and is the most likely carrier of electrons destined for outer surface during respiration on solid metal oxides, a process that requires extracellular electron transfer. This cytochrome has the highest content of lysine residues (24%) among the family, and it was suggested to be involved in e^−^/H^+^ energy transduction processes. In the present work, we investigated the functional role of lysine residues strategically located in the vicinity of each heme group. Each lysine was replaced by glutamine or glutamic acid to evaluate the effects of a neutral or negatively charged residue in each position. The results showed that replacing Lys^9^ (located near heme IV), Lys^18^ (near heme I) or Lys^22^ (between hemes I and III) has essentially no effect on the redox properties of the heme groups and are probably involved in redox partner recognition. On the other hand, Lys^43^ (near heme IV), Lys^52^ (between hemes III and IV) and Lys^60^ (near heme III) are crucial in the regulation of the functional mechanism of PpcA, namely in the selection of microstates that allow the protein to establish preferential e^−^/H^+^ transfer pathways. The results showed that the preferred e^−^/H^+^ transfer pathways are only established when heme III is the last heme to oxidize, a feature reinforced by a higher difference between its reduction potential and that of its predecessor in the order of oxidation. We also showed that K43 and K52 mutants keep the mechanistic features of PpcA by establishing preferential e^−^/H^+^ transfer pathways at lower reduction potential values than the wild-type protein, a property that can enable rational design of *Gs* strains with optimized extracellular electron transfer capabilities.

## Introduction

*Geobacter* species are of special interest because of their capability of transferring electrons to extracellular substrates during anaerobic respiration on insoluble terminal electron acceptors. This feature is currently being explored in developing *Geobacter*-based biotechnological applications, particularly in the fields of bioremediation and bioenergy, such as removal of toxic or radioactive metals and electricity production by microbial fuel cells [1]–[3]. The detailed comparative analysis of the genomes of six *Geobacter* species (*G. bemidjiensis, G. strain FRC-32, G. lovleyi, G. metallireducens, G. sulfurreducens and G. uraniireducens*) showed that, except for *G. lovleyi*, the most remarkable feature is the presence of a large number and diversity of *c*-type cytochromes [4]–[7]. However, most cytochromes are poorly conserved among the *Geobacter* species [4]. One noticeable exception to this general feature is the family of triheme periplasmic cytochromes *c*_7_ present in all species of *Geobacter*. The triheme cytochromes from *G. sulfurreducens* (*Gs*) is the only family studied in detail to date by genetic, functional and structural methods [8]–[18]. This family consists of five cytochromes, designated PpcA-E with approximately 10 kDa molecular weight [14]. Each protein contains three low-spin heme groups with bis-histidinyl axial coordination and a conserved overall fold [11], [18]. The functional electron transfer mechanisms of *Gs* triheme cytochromes have been characterized in detail, except for PpcC due to the presence of multiple conformers during the redox cycle of this protein [19]. The results obtained suggested that PpcA and PpcD can couple e^−^/H^+^ transfer, a property that might contribute to the generation of a proton electrochemical gradient across the periplasmic membrane [17], [20]. Interestingly, PpcA and PpcD are the only members of the family whose deletion has significantly altered *Gs* phenotype with respect to the reduction of iron oxides [15], [16].

**Figure 1:**
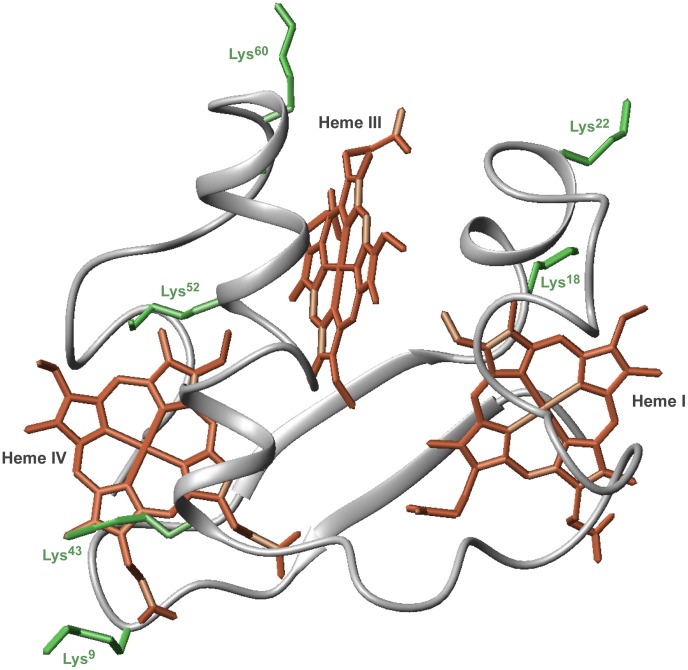
Spatial location of lysine residues mutated in this work depicted in PpcA solution structure (PDB code, 2LDO [11]). The PpcA polypeptide chain (gray) is shown as C_α_ ribbon and heme groups (red). The side chain of Lys^9^, Lys^18^, Lys^22^ Lys^43^, Lys^52^ and Lys^60^ (green) are represented as stick drawings. The hemes are numbered I, III and IV, a designation that derives from the superimposition of the hemes in cytochromes *c*_7_ with those of the structurally homologous tetraheme cytochromes *c*_3_.

A salient feature of *Gs* triheme cytochromes is their high lysine content as shown in Figure S1. This feature is expected to have a role in the modulation of the functional properties of these cytochromes, as well as in the recognition of redox partners. Amongst this family, PpcA is the most abundant cytochrome in the *Gs* periplasm and has the highest content of lysine (24%). In addition to its crystal structure [14], the solution structure of PpcA was determined [11] and can be used for the rational design of PpcA mutants. In the present work, we aimed to probe the functional role of selected lysine residues in the vicinity of each heme group in PpcA. The spatial localization of the selected lysine residues in PpcA structure is depicted in Figure 1: lysine 18 (near heme I), lysine 22 (between hemes I and III), lysine 60 (near heme III) and lysines 9, 43 and 52 (near heme IV). Each lysine was substituted by glutamine and glutamic acid to evaluate the effects of replacing the positively charged amino acid at each position by a neutral or negatively charged residue. For each of the twelve mutants, the redox properties were first screened at pH 6 and pH 8. For those mutants showing clear differences in redox behavior compared to the wild-type cytochrome, the detailed thermodynamic properties and the functional electron transfer mechanisms were determined using visible and NMR spectroscopy. The comparison of the redox properties of the mutants with those of the wild-type cytochrome allowed us to elucidate the role of the selected lysine residues in the functional mechanism of PpcA.

## Materials and Methods

### Site directed mutagenesis

For mutagenesis, QuikChange Site-Directed Mutagenesis Kit (Stratagene) was used in accordance with the manufacturer's instructions. Oligonucleotides were synthesized by MWG Biotech (High Point, NC). PpcA expression vector pCK32 [21] was used as a template. The presence of desired mutations was confirmed by DNA sequencing performed by MWG Biotech.

### Bacterial growth and purification of PpcA mutants

*Escherichia coli* strain BL21(DE3) containing the plasmid pEC86 [22] was transformed with the expression vector containing each of the PpcA mutant sequences (K9Q/E, K18Q/E, K22Q/E, K43Q/E, K52Q/E and K60Q/E). Transformed *E. coli* cells were grown in 2xYT medium containing 34 µg/mL chloramphenicol and 100 µg/mL ampicillin. Protein expression was induced by adding isopropyl-β-D-thiogalactoside (IPTG) to a final concentration of 10 µM. PpcA mutants were purified by cation exchange and gel filtration, as described for the wild-type cytochrome [14]. The purity of the proteins was evaluate by SDS-PAGE (15%), stained with Coomassie blue. To confirm the correct heme incorporation, electrospray ionization mass spectrometry was performed at the HHMI Biopolymer Laboratory and W.M. Keck Foundation Biotechnology Resource Laboratory at Yale University.

### NMR studies

#### Preparation of NMR samples

NMR samples and experimental conditions matched those used in the characterization of the wild-type protein in the reduced and intermediate state of oxidation [17] and are summarized below. PpcA lysine mutant samples of about 140 µM for fully reduced studies and 70 µM for redox titrations were prepared from lyophilized protein in 80 mM phosphate buffer with NaCl (250 mM final ionic strength) in D_2_O. Full reduction of the samples was achieved by adding gaseous hydrogen in the presence of catalytic amounts of the enzyme Fe-hydrogenase, from *Desulfovibrio vulgaris* (Hildenborough). Partially oxidized samples were obtained by first removing the hydrogen from the reduced sample with argon followed by the addition of controlled amounts of air into the NMR tube. The pH of the samples at the reduced and intermediate stages of oxidation was adjusted inside an anaerobic glove chamber with argon circulation to avoid sample reoxidation.

#### NMR experiments and assignment

All the NMR experiments were performed on a Bruker Avance 600 spectrometer equipped with a triple-resonance cryoprobe (TCI) at 288K. As in the case of the wild-type cytochrome [17], NMR spectra were obtained before and after the lyophilization of each mutant to confirm that the protein integrity was not affected. NMR experiments for the fully reduced samples were acquired in the same experimental conditions to those of wild-type protein [10], [23]. The following experiments were acquired: 2D-^1^H TOCSY (45 ms) and 2D-^1^H-NOESY (100 ms). To monitor the stepwise oxidation of the individual hemes in PpcA lysine mutants, 2D-^1^H-exchange spectroscopy (EXSY) spectra in the pH range 6–9 were acquired in partially oxidized samples, as previously described for the wild-type protein [17]. Spectra were processed using TOPSPIN (BrukerBiospin, Karlsruhe, Germany). The program Sparky - NMR Assignment and Integration Software [24] was used for inspection and for assignment of the signals in the NMR spectra. The assignment of specific heme signals to the corresponding hemes in the reduced state of PpcA lysine mutants was performed using the methodology previously used for the wild-type protein [10], [17].

### Redox titrations followed by visible spectroscopy

Due to the negative reduction potentials of the redox centres, PpcA lysine mutant redox titrations followed by visible spectroscopy, were carried out inside an anaerobic chamber (MBraun) as described previously [10]. Samples with 18 µM protein concentration were prepared at pH 7 and 8 in NaCl/phosphate buffer solution with a final ionic strength of 250 mM. Redox titrations were carried out at 288 K. The reduced fraction of the proteins was determined by integrating the area of the α-peak (551 nm) above the line connecting the flanking isosbestic points (543 and 559 nm) to subtract the optical contribution of the redox mediators, as described for the wild-type protein [10]. To check for hysteresis, each redox titration was performed in both oxidative and reductive directions. The experiments were performed at least two times, and the reduction potentials (relative to standard hydrogen electrode, SHE) were found to be reproducible within ± 5 mV.

### Thermodynamic model

In the case of a triheme cytochrome, three consecutive reversible steps of one-electron transfer convert the fully reduced state (stage 0, *S_0_*) to the fully oxidized state (stage 3, *S_3_*), and therefore four different redox stages can be defined, each group consisting of microstates with the same number of oxidized hemes (see Fig. S2), as previously described for the wild-type protein [17]. The general theoretical framework that allows the detailed study of the redox centre thermodynamic properties in multiheme proteins [25] was used to determine the thermodynamic parameters of the redox centres for the wild-type protein [17] and is summarised below for clarity. In summary, taking as reference the fully reduced and protonated protein, the energy of the microstates can be described in the full range of pH and solution potential as sums of 10 parameters: the three energies of oxidation of the hemes (reduction potentials), the *pK_a_* of the redox-Bohr centre, plus six two-centre interactions energies (three heme-heme and three redox-Bohr interactions). To obtain such information it is necessary to monitor the stepwise oxidation of each heme using 2D-^1^H EXSY NMR experiments, which allows discriminating the individual heme signals in different oxidation stages [26], [27]. The information obtained from the NMR data allows the determination of the thermodynamic parameters relative to reference state (for a review see [25]).

In the characterization of the wild-type protein the chemical shifts obtained for the heme methyls 12^1^CH_3_^I^, 7^1^CH_3_^III^, 12^1^CH_3_^IV^ (labelled according to the IUPAC nomenclature – see Figure S3) in each oxidation stage in the pH range 6–9 were fitted simultaneously with visible redox titrations data obtained at pH 7 and 8.

In order to obtain thermodynamic parameters of the PpcA lysine mutants the same set of heme methyls were used and visible redox titrations were performed in identical experimental conditions. The experimental uncertainty of the NMR data was evaluated from the line width of each NMR signal at half height; the visible data points were given an uncertainty of 3% of the total optical signal.

## Results and Discussion

### Impact of the mutations on the cytochromes global fold and heme core

The yields of the twelve PpcA mutants were similar to that obtained for the wild-type protein (approximately 3 mg per liter of culture). The mutants showed identical UV-visible and NMR spectroscopic features both in the reduced and oxidized states compared to the wild-type protein (data not shown).

Due to the small size of triheme cytochrome PpcA (71 residues), the core of the protein formed by the three heme groups is essentially covered by the polypeptide chain in an approximate ratio of 24 residues per heme group. Moreover, due to the strong ring-current effects produced by the heme groups, the proton chemical shifts of the heme substituents are determined by the relative orientation of the hemes and neighbouring residues. Therefore, heme proton chemical shifts provide an excellent probe to detect major changes in the overall fold of the polypeptide chain and to evaluate the impact of the substitution of each lysine by glutamine or glutamic acid. The impact of the mutations on the heme core architecture was probed by 2D-^1^H NMR. The heme proton resonances were assigned using the same methodology described for PpcA [10] and their chemical shifts were compared to those of wild-type cytochrome (see Table S1 and Fig. S4). The rmsd values calculated from these chemical shifts are low, though, as expected slightly higher for the heme signals that are closer to the mutated residue. The small values of rmsd and, therefore, the good correlation obtained for the chemical shifts of heme protons in the native and mutated cytochromes, indicate that the heme core of the proteins were unaffected by the mutation. In addition, the same set of NOE connectivities between the heme groups were observed for native and mutant proteins, further confirming that the heme core arrangement is similar in all the proteins.

### In silico modeling of mutations

Side chains of lysine residues that are fully exposed to solvent are usually not well-defined and can have many different conformations resulting from rotations around single bonds. However, in less exposed lysine side chains their rotational freedom can be restricted by the surroundings. In the solution structure of PpcA, for some of the lysine residues, like Lys^9^, many conformations were observed, while for Lys^43^ only a few conformations were observed (PDB code, 2LDO [11]). The distance between the well-defined C_β_ of the selected lysine residues to the nearest iron(s) in the heme(s) is shown in Table 1 (see also Fig. 1).

**Table 1:** Distance between the C_β_ of selected lysine residues to the nearest heme iron(s) in the solution structure of PpcA (PDB code, 2LDO [11]).

	Distances (Å)		
	Heme I	Heme III	Heme IV
Lys^9^		21.2	10.4
Lys^18^	9.6	11.0	
Lys^22^	12.7	12.0	
Lys^43^		16.0	6.4
Lys^52^		8.4	7.6
Lys^60^	18.4	7.9	

To determine whether the difference in side chains can alter the local structure, the lysine residues were replaced *in-silico* by glutamic acid. The modeling studies show that the surface of lysines 9, 18, 22 and 60 can be replaced by glutamic acid (or glutamine) without any structural alterations. Even replacing the partially buried lysines 43 and 52 by glutamic acid did not indicate any steric clashes that cannot be avoided by slight adjustment of the standard side chain rotamers. Therefore, it can be concluded that the change observed in redox properties of mutants K43, K52 and K60 (see below) are primarily due to replacement of the positive charge at that position.

### Screening the effect of the mutations on the relative heme oxidation fractions

The heme oxidation fractions in the wild-type cytochrome were previously determined from the chemical shifts of the heme methyls 12^1^CH_3_^I^, 7^1^CH_3_^III^, 12^1^CH_3_^IV^ measured in the different oxidation stages from 2D-^1^H EXSY NMR experiments, according to the methodology summarized in the Materials and Methods section. Since PpcA exhibits fast intramolecular and slow intermolecular electron exchange on the NMR time scale, the individual heme signals in different oxidation stages can be discriminated and used to monitor the oxidation profiles of the hemes (for a review see [13], [17]). The data obtained for PpcA showed that the heme oxidation fractions are modulated by the solution pH in the region 6–8. This modulation, known as redox-Bohr effect, is observed in the physiological pH range for *G. sulfurreducens* cellular growth giving the protein the necessary properties to couple e^−^/H^+^ transfer [17], [20]. Thus, in the present work we first screened the oxidation profiles of the individual hemes in each mutant at pH 6 and 8 by measuring the chemical shifts of heme methyls 12^1^CH_3_^I^, 7^1^CH_3_^III^, 12^1^CH_3_^IV^ in the different oxidation stages. This is illustrated in the 2D-^1^H EXSY NMR spectra obtained at pH 8 for each mutant in Figure S5. Such strategy allowed us to identify and select the mutants with considerable differences in their heme oxidation profiles compared to the wild-type for further detailed thermodynamic characterization.

**Figure 2:**
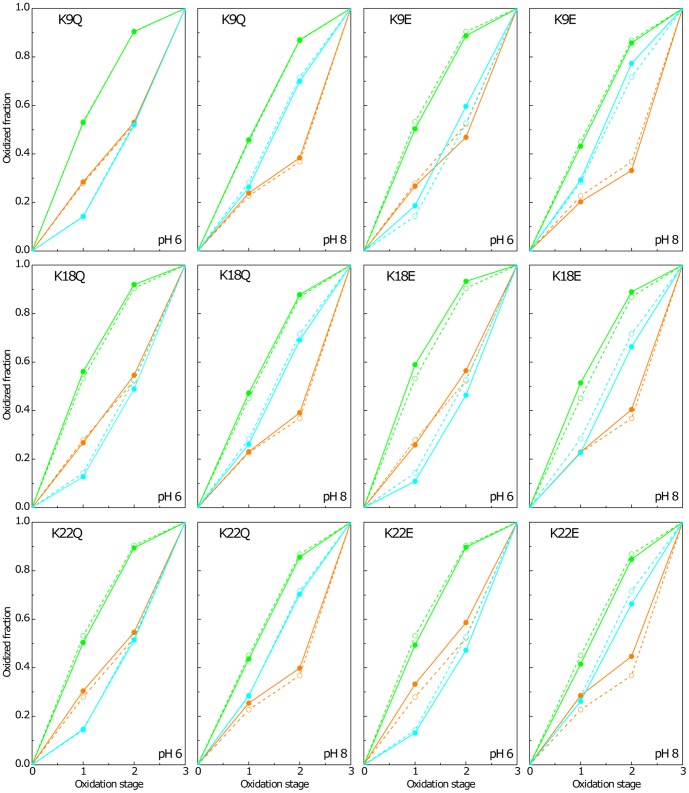
Oxidation fraction of K9, K18 and K22 PpcA mutants (solid symbols and lines) and PpcA (open symbols and dashed lines) at pH 6 and pH 8. Data for hemes I, III and IV are colored in green, orange and blue, respectively. The heme oxidation fractions were calculated according to equation *x_i_*  =  (δ_i_-δ_0_)/(δ_3_-δ_0_), where δ_i_, δ_0_, and δ_3_ are the observed chemical shifts of each methyl in stage i, 0, and 3, respectively.

**Figure 3:**
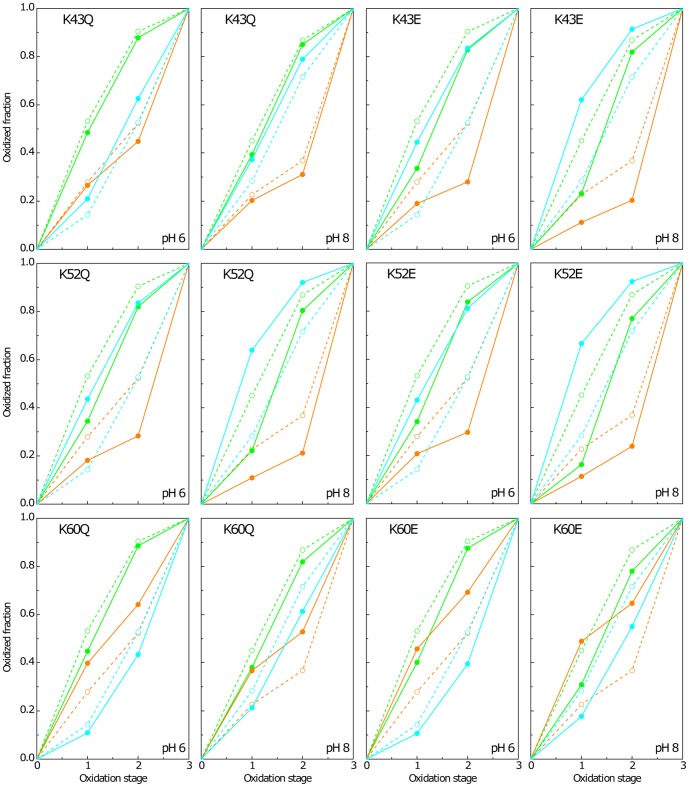
Oxidation fraction of K43, K52 and K60 PpcA mutants (solid symbols and lines) and PpcA (open symbols and dashed lines) at pH 6 and pH 8. Data for hemes I, III and IV are colored in green, orange and blue, respectively. The heme oxidation fractions were calculated as described in legend of Fig. 2.

The oxidation pattern of individual hemes of the mutants and wild-type proteins obtained at pH 6 and 8 are indicated in Figure 2 and 3. The comparison between the heme oxidation fractions, at both pH values, shows that the heme oxidation profiles were only slightly altered for K9, K18 and K22 but significantly changed for K43, K52 and K60 (*cf.* dashed and solid lines in Fig. 2 and 3). In the latter group of mutants (K43, K52 and K60) two main features emerge from the analysis of their oxidation patterns: (i) the hemes located near the replaced residue are the most affected ones (heme III for K60 and heme IV for K43 and K52 mutants) and (ii) the more affected hemes have higher oxidation fraction values compared to the wild-type protein (*cf.* dashed and solid lines in Fig. 3). This latter observation can be rationalized on a pure electrostatic basis. Indeed, the replacement of a positively charged lysine with a neutral glutamine or with a negatively charged glutamic acid is expected to stabilize the oxidized form of the nearest heme by lowering its reduction potential and, concomitantly, increase its oxidation fraction, compared to the other heme groups. Since K43, K52 and K60 mutants showed significant differences in their oxidation profiles compared to the wild-type protein, their functional properties were characterized in detail in the present work.

### Redox characterization of heme groups and redox-Bohr centre in K43, K52 and K60 mutants

**Figure 4:**
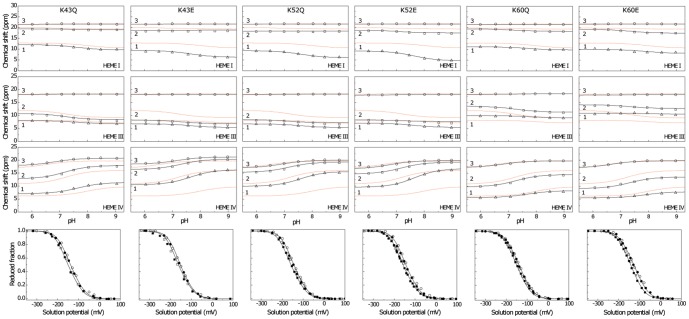
Fitting of the thermodynamic model to the experimental data for PpcA mutants. The black solid lines are the result of the simultaneous fitting of the NMR and visible data. The three upper panels show the pH dependence of heme methyl chemical shifts at oxidation stages 1 (▵), 2 (□), and 3 (○). The lower panel corresponds to the reduced fractions determined by visible spectroscopy at pH 7 (○) and pH 8 (□). The open symbols and the filled symbols represent the data points in the reductive and oxidative titrations, respectively. The solid red lines in each panel represent the best fit for the wild-type protein [17].

In order to probe the functional role of each lysine residue in the modulation of the redox properties of PpcA, the detailed thermodynamic characterization of the six selected mutants (K43Q, K43E, K52Q, K52E, K60Q and K60E) was carried out. The thermodynamic parameters of the wild-type protein were previously determined by fitting the pH dependence of the chemical shifts of heme methyls 12^1^CH_3_^I^, 7^1^CH_3_^III^ and 12^1^CH_3_^IV^, measured in different stages of oxidation, together with data from visible redox titrations obtained at pH 7 and 8, within the framework of an electrostatic model that considers four interacting centres: three hemes and one protonatable centre [17]. In the present work, the same set of heme methyl groups were used to characterize the redox properties of PpcA mutants. 2D-^1^H EXSY NMR spectra were collected at different pH values and the chemical shifts of heme methyls 12^1^CH_3_^I^, 7^1^CH_3_^III^ and 12^1^CH_3_^IV^ were measured for oxidation stages 1–3 (Fig. 4). As for the fully reduced form (Table S1), in the fully oxidized state (stage 3) the chemical shifts of the heme methyls from PpcA mutants are little affected (*cf*. corresponding black and red solid lines in Fig. 4). On the other hand, at intermediate oxidation stages (stages 1 and 2) a different scenario is observed. In this case, the chemical shifts of the heme methyls are quite different (Fig. 4). The most affected heme methyl in K43Q/E and K52Q/E is 12^1^CH_3_^IV^, whereas in K60Q/E is 7^1^CH_3_^III^. In each case, the most affected heme is located nearest to the mutated residue in the structure (see Fig. 1). By having essentially unaffected chemical shifts in the fully reduced and fully oxidized forms, the variations on the chemical shifts at intermediate oxidation stages reflect important changes in the heme oxidation fractions, *i.e.* changes in their redox parameters, during the oxidation of the proteins (see Fig. 4). It is important to note that in each oxidation stage, the number of oxidized hemes is fixed, *i.e*. one in oxidation stage 1 and two in oxidation stage 2 (Figure S2 – see Materials and Methods). Thus, if one particular heme is more oxidized, the others are necessarily less oxidized. This is clearly illustrated in the oxidation pattern of the heme groups at pH 6 and 8 indicated in Figure 2 and 3.

In order to quantify the effect of the mutations on the redox properties of the heme groups, the thermodynamic model was fit to the pH dependence of the observed chemical shift of heme methyls, together with data from visible redox titrations (see Materials and Methods). The thermodynamic parameters determined for the six mutants are indicated in Table 2. The quality of the fittings obtained for the pH dependence of the paramagnetic chemical shifts and for the visible redox titrations clearly shows that the experimental data is well described by the model (see black solid lines in Fig. 4). In the six mutants, the redox and redox-Bohr interactions are weaker compared to those of PpcA (Table 2). However, as observed for PpcA, the smallest redox interactions are established between the hemes that are structurally further apart (hemes I and IV) and the strongest redox-Bohr interactions with heme IV. The lower values of the redox interactions, in the context of a conserved heme core, might have their origin on structural rearrangements of charged groups that cause variations in local dielectric constants [28]. The positive values of the redox interactions indicates that the oxidation of a particular heme renders the oxidation of its neighbors more difficult, which is expected on an electrostatic basis. In fact, the oxidation of one particular heme, *i.e.* transfer of one electron, decreases the number of negative charges in the vicinity of its neighbor, stabilizing the reduced form of the latter. Similarly, the negative redox-Bohr interactions (between the hemes and the redox-Bohr centre) indicate that the oxidation of the hemes facilitates the deprotonation of the redox-Bohr centre and vice-versa. The higher redox-Bohr interaction observed with heme IV in the mutants, indicates that the redox-Bohr centre remains associated with heme IV, as observed in the wild-type protein [10], [11], [17]. In relation to the heme reduction potentials, the most affected ones were those of hemes III (K60Q/E) and IV (K43Q/E and K52Q/E). In all cases the reduction potential in the fully reduced and protonated proteins decreased, as expected from the removal of a positive charge in the mutant proteins from the neighborhood of the affected heme groups. The observed variations suggest an important role for residues Lys^43^, Lys^52^, and Lys^60^ in the regulation of the redox properties of hemes III and IV.

**Table 2:** Thermodynamic parameters for PpcA mutants.

	Energy (meV)			
Protein	Heme I	Heme III	Heme IV	Redox-Bohr centre
**K43Q**				
Heme I	**−161 (5)**	20 (2)	6 (3)	−17 (4)
Heme III		−**146 (4)**	30 (3)	−15 (4)
Heme IV			−**141(5)**	−42 (4)
Redox-Bohr centre				**463 (8)**
**K43E**				
Heme I	−**161 (3)**	16 (2)	−3 (2)	−11 (3)
Heme III		−**145 (3)**	20 (2)	−6 (3)
Heme IV			−**167 (3)**	−38 (3)
Redox-Bohr centre				**463 (6)**
**K52Q**				
Heme I	−**154 (3)**	20 (2)	0 (2)	−15 (3)
Heme III		−**138 (3)**	20 (2)	−11 (3)
Heme IV			−**160 (4)**	−43 (3)
Redox-Bohr centre				**466 (6)**
**K52E**				
Heme I	−**154 (3)**	20 (2)	−1 (2)	−14 (3)
Heme III		−**142 (3)**	27 (2)	−22 (3)
Heme IV			−**160 (4)**	−59 (3)
Redox-Bohr centre				**482 (6)**
**K60Q**				
Heme I	−**164 (3)**	22 (2)	6 (2)	−15 (3)
Heme III		−**162 (3)**	30 (2)	−16 (3)
Heme IV			−**132(3)**	−40 (3)
Redox-Bohr centre				**461 (6)**
**K60E**				
Heme I	−**151 (5)**	24 (3)	9 (3)	−23 (5)
Heme III		−**157 (6)**	35 (3)	−29 (5)
Heme IV			−**121 (6)**	−49 (5)
Redox-Bohr centre				**482 (11)**
**PpcA**				
Heme I	−**154 (5)**	27 (2)	16 (3)	−32 (4)
Heme III		−**138 (5)**	41 (3)	−31 (4)
Heme IV			−**125 (5)**	−58 (4)
Redox-Bohr centre				**495 (8)**

For comparison the values previously obtained for PpcA [17] were also included. For each cytochrome, the fully reduced and protonated protein was taken as reference. Diagonal values (in bold) correspond to oxidation energies of the hemes and deprotonating energy of the redox-Bohr centre. Off-diagonal values are the redox (heme-heme) and redox-Bohr (heme-proton) interactions energies. All energies are reported in meV, with standard errors given in parenthesis.

### Impact of the mutations on the heme oxidation order at physiological pH

**Figure 5:**
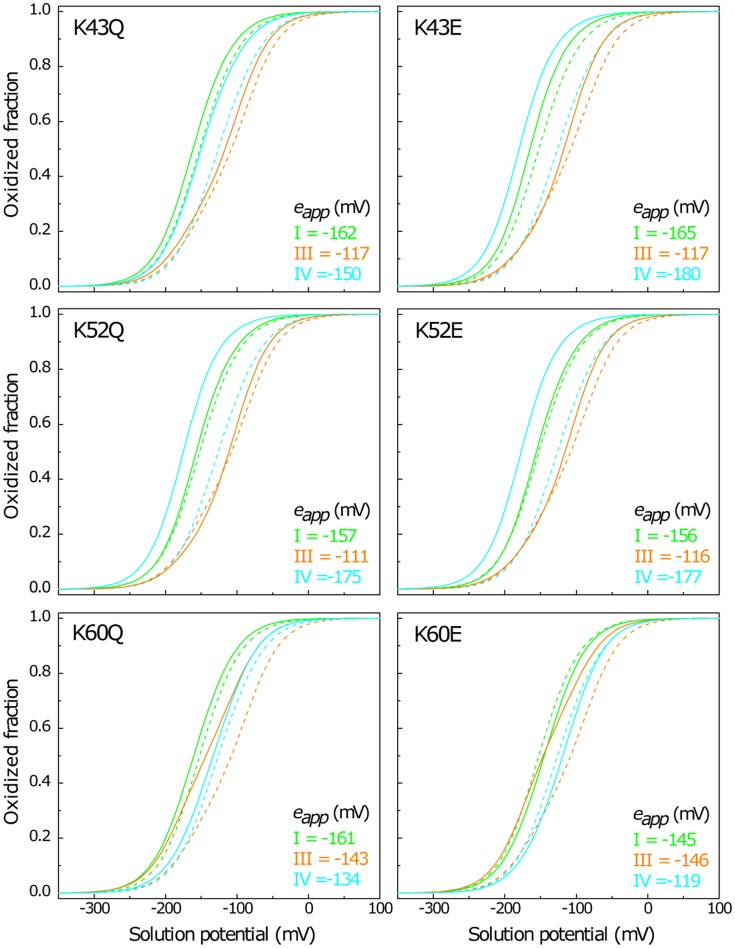
Oxidized fractions of the individual hemes for PpcA mutants (solid lines) and wild-type (dashed lines) at pH 7.5. The curves were calculated as a function of the solution reduction potential using the parameters listed in Table 2. The midpoint reduction potentials of the hemes (*e_app_*) are also indicated.

As shown above, the reduction potential of each heme group is affected by the oxidation state of neighboring hemes (redox interactions) and by the solution pH (redox-Bohr interactions). To evaluate the effect of the mutations on the heme midpoint reduction potentials (*e_app_, i.e.* the point at which the oxidized and reduced fractions of each heme are equally populated) at physiological pH, the oxidation curves of the individual hemes for K43, K52 and K60 mutants were computed from the thermodynamic parameters listed in Table 2 (Fig. 5 and Table 3). As for the wild-type protein, the redox interactions modulate the electron affinity of each heme so that the curves are non-Nernstian, showing some cross over during the redox titration (Fig. 5). The heme oxidation profiles of the mutants are different compared to the wild-type protein (*cf*. solid and dashed lines in Fig. 5), which reflects the differences in the redox parameters and particularly in their heme reduction potentials. From the analysis of the individual oxidation profiles of PpcA mutants it is clear that at physiological pH, the hemes located in the proximity of the mutated residue are the most affected ones, *i.e.*, heme III for K60 and heme IV for K43 and K52 mutants. Compared to the wild-type the *e_app_* values are smaller, as expected from the replacement of a positive charge in their vicinity. A significant decrease in the reduction potential is observed for heme IV (34/54 mV and 49/51 mV for K43Q/E and K52Q/E mutants, respectively). The significant decrease in the reduction potential of heme IV in K43E and K52Q/E mutants alters the heme oxidation order in these proteins (IV-I-III) compared to the wild-type (I-IV-III). The difference between the *e_app_* values of the first two hemes (heme IV and I) is of the same order of magnitude compared to that observed for the wild-type protein (Table 3). However, the difference between the *e_app_* values of the second and third heme to be oxidized is considerable higher (Table 3). On the other hand, the replacement of the positively charged lysine at position 60 decreases significantly the *e_app_* value of heme III (35 mV and 38 mV for K60Q and K60E, respectively) compared to the wild-type. Therefore, the oxidation curve of heme III is shifted to more negative values towards those of the other hemes (Fig. 5). As a consequence, in these proteins heme III is no longer the last one to be oxidized so that the heme oxidation order is I-III-IV for K60Q and (III,I)-IV for K60E.

**Table 3:** Comparison of the results obtained from the thermodynamic characterization of PpcA mutants at pH 7.5.

Protein	*e_app_* (mV)			Order of heme oxidation	Δ*e_app_* (mV) (2^nd^/1^st^)	Δ*e_app_* (mV) (3^rd^/2^nd^)	Electron transfer pathway
	Heme I	Heme III	Heme IV				
PpcA	−152	−108	−126	I-IV-III	26	18	*P_0H_* → *P_1H_* → *P_14_* → *P_134_*
K43Q	−162	−117	−150	I-IV-III	12	33	*P_0H_* →(*P_1H_*)→ *P_14_* → *P_134_*
K43E	−165	−117	−180	IV-I-III	15	48	*P_0H_* → *P_14_* → *P_134_*
K52Q	−157	−111	−175	IV-I-III	18	46	*P_0H_* → *P_14_* → *P_134_*
K52E	−156	−116	−177	IV-I-III	21	40	*P_0H_* → *P_14_* → *P_134_*
K60Q	−161	−143	−134	I-III-IV	18	9	No preferential pathway
K60E	−145	−146	−119	(III,I)-IV	1	26	No preferential pathway
PpcB	−150	−155	−130	(III,I)-IV	5	20	No preferential pathway
PpcD	−156	−102	−162	IV-I-III	6	54	*P_0H_* → *P_14_* → *P_134_*
PpcE	−158	−158	−100	(III,I)-IV	0	58	No preferential pathway
M58S	−159	−110	−139	I-IV-III	20	29	*P_0H_* → *P_1H_* → *P_14_* → *P_134_*
M58D	−160	−139	−140	I-(III,IV)	20	1	No preferential pathway
M58K	−159	−91	−146	I-IV-III	13	55	*P_0H_* → *P_14_* → *P_134_*
M58N	−163	−90	−152	I-IV-III	11	62	*P_0H_* → *P_14_* → *P_134_*

For comparison the values previously obtained for PpcA, PpcB, PpcD and PpcE [17] and PpcAM58 mutants [29] were also included. Δ*e_app_* (2^nd^/1^st^) is the difference between the *e_app_* values of the second and the first heme to oxidized. Δ*e_app_* (3^rd^/2^nd^) is the difference between the *e_app_* values of the third and second heme to oxidized.

### Role of K43, K52 and K60 on the functional mechanism of PpcA

**Figure 6:**
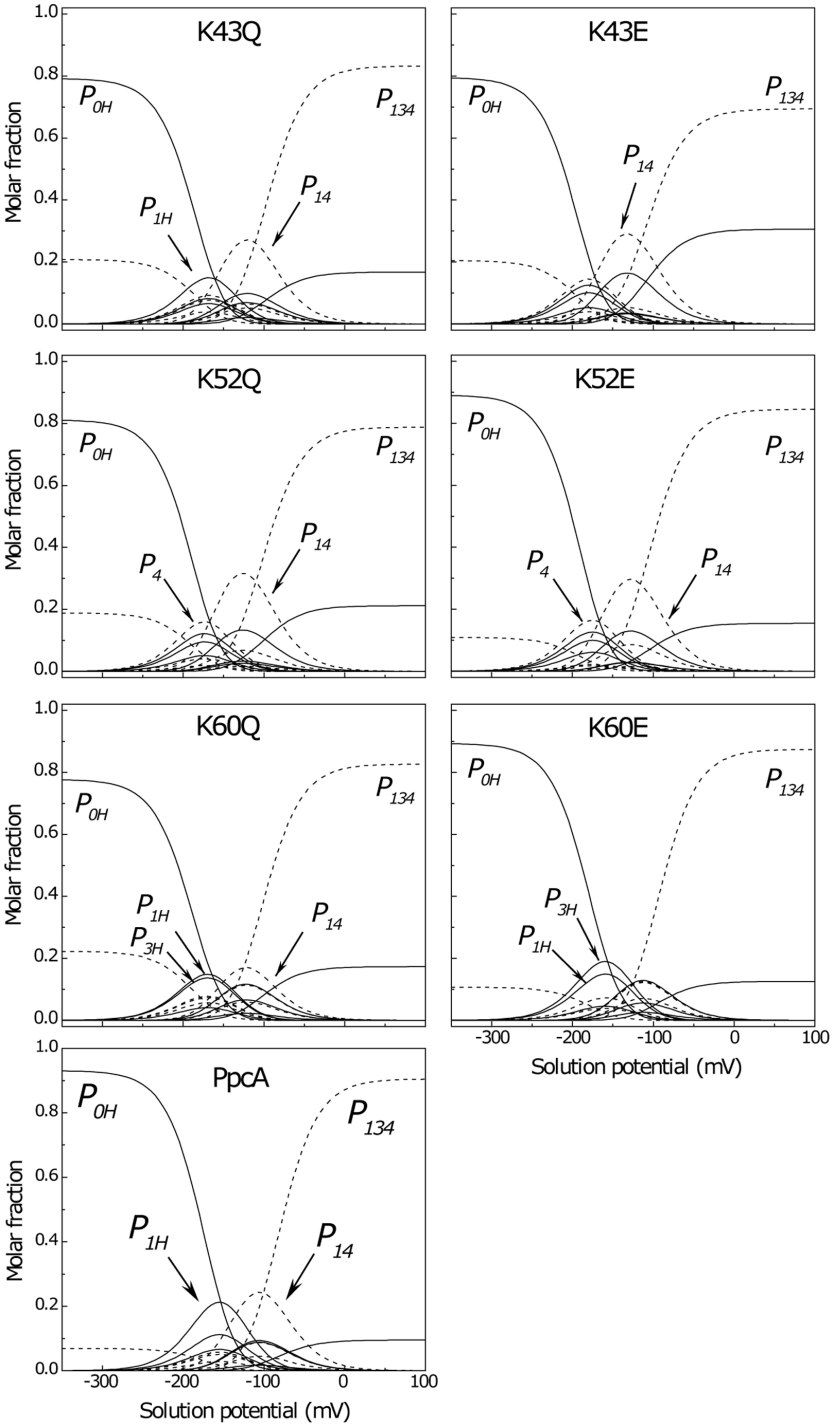
Molar fractions of the 16 individual microstates (described in Fig. S2) of PpcA mutants and the wild-type cytochrome at pH 7.5. The curves were calculated as a function of the solution reduction potential using the parameters listed in Table 2. Solid and dashed lines indicate the protonated and deprotonated microstates, respectively. For clarity only the relevant microstates for a given mutant are labelled.

As shown above, the oxidation profile of the redox centres is highly dependent on the nature of the side chain at positions 43, 52 or 60. Thus, to evaluate the effect of each mutation on PpcA functional mechanism, the relative contribution of the 16 possible microstates (see Fig. S2) was determined as function of the solution potential for K43, K52 and K60 mutants (Fig. 6). Such studies were previously undertaken for the wild-type protein and a coherent electron transfer pathway coupled to proton transfer was established [17]. The relative variation of the microstates in PpcA is also depicted in Figure 6 together with those obtained in the present work for K43, K52 and K60 mutants. While in the wild-type protein a concerted e^−^/H^+^ transfer occurs between oxidation stages 1 and 2, it is clear that the relative contributions of different microstates to the overall population in each stage are significantly altered in the lysine mutants (Fig. 6). In fact, in the wild-type cytochrome the oxidation stage 0 is dominated by the fully reduced and protonated form *P_0H_* and stage 1 is dominated by the oxidation of heme I (*P_1H_*) while keeping the redox-Bohr centre protonated. Oxidation stage 2 is dominated by the oxidation of hemes I and IV and deprotonation of the acid-base centre (*P_14_*), that remains deprotonated in stage 3 upon full oxidation of heme III (*P_134_*). Therefore, a route is defined for electron transfer in PpcA: *P_0H_* → *P_1H_* → *P_14_* → *P_134_*. This is not the case for the K60 mutants for whom the lowering of the *e_app_* of heme III brings the midpoint reduction potential values of all hemes groups closer to each other and favours the oxidation of this heme at lower reduction potentials (*cf.* dashed and solid lines on Fig. 5). Consequently, for the K60 mutants, two microstates (*P_1H_* and *P_3H_*) are now dominant in the first oxidation stage and, thus, no preferential pathway for electron transfer is observed (Fig. 6). On the other hand, for K43 and K52 mutants a different scenario is observed. As mentioned above, the major effect of replacing the positive charge is the lowering of the *e_app_* values of heme IV. Consequently, this heme is more oxidized in the first oxidation stage, compared to PpcA. In the case of K43E, K52Q and K52E the significant reduction on the *e_app_* of heme IV alters the oxidation order of the hemes (see Fig. 6 and Table 3) so that heme IV is now the first heme to oxidize. In all these mutants heme III is clearly the last one to oxidize. Therefore, and since the *e_app_* of heme I is closer to that of heme IV (*cf.* solid green and blue lines in Fig. 5) the microstate with heme I and IV oxidized (*P_14_*) in the mutants has a much higher population compared to the wild-type protein (Fig. 6). In the mutants, the oxidation stage 0 is also dominated by the protonated form *P_0H_*, but the microstates of oxidation stage 1 are overcome by the *P_14_* curve, which intercepts earlier the curve of microstate *P_0H_*. The microstate *P_14_* dominates the oxidation stage 2, whereas *P_134_* dominates stage 3. Thus, in these three mutants, a different preferential route for electrons is established, favoring a proton-coupled 2e^−^ transfer step between oxidation stages 0 and 2: *P_0H_* → *P_14_* → *P_134_* as observed for PpcD and M58K and M58N mutants [17], [29].

Taken these observations together, it is clear that residues Lys^43^, Lys^52^ and Lys^60^ modulate the reduction potential of their closest heme, which in turn controls the microscopic redox states that can be accessed during the redox cycle of the proteins. However, the repercussion of the substitution of a positive charge close to heme III (Lys^60^ mutants) or IV (Lys^43^ and Lys^52^ mutants) has distinct effects on the functional mechanism of the protein. In case of Lys^60^, the replacement of the positive side chain by the neutral or negatively charged one altered the reduction potential of heme III in a way that it is no longer the last heme to oxidize. In this case, no preferential pathway for e^−^/H^+^ transfer is observed. In contrast, in the case of Lys^43^ and Lys^52^ mutants, the removal of a positively charged side chain either at position 43 or 52 lead to a higher stabilization of the oxidized form of heme IV, compared to the other two hemes, and a different functional mechanism emerges in which a 2e^−^ step coupled with proton transfer is observed. This suggests that the reduction potential of heme III relative to the other two hemes seems to be crucial in enabling these proteins to couple electron transfer with deprotonation of the redox-Bohr centre. Indeed, only when heme III is the last one to oxidize (higher *e_app_* value) preferential e^−^/H^+^ pathways are established, which are reinforced by a higher separation between the *e_app_* values of the second and third hemes to oxidize (see Table 3). However, the pathway varies with the separation between the *e_app_* values of heme III and its predecessor in the order of oxidation (Table 3). In the case of PpcA such separation was 18 mV and the route for electron transfer was: *P_0H_* → *P_1H_* → *P_14_* → *P_134_*. In case of K43Q the same route was observed but the slightly higher separation between the *e_app_* values (33 mV *versus* 18 mV in the wild-type) increased the contribution of microstate *P_14_*. Finally, in K43E, K52Q and K52E the higher separation between the *e_app_* values of hemes III and I (48, 46 and 40 mV, respectively) lead to a significant contribution of the microstate *P_14_* so that a different preferred route for electrons was observed: *P_0H_* → *P_14_* → *P_134_*. Interestingly, the results obtained from the thermodynamic characterization of the *G. sulfurreducens* PpcA family members (PpcA, PpcB, PpcD, and PpcE) [17], as well as PpcA mutants of residue Met^58^, a residue that controls the solvent accessibility of heme III [29], have shown that preferential pathways for e^−^/H^+^ coupling were established only when heme III has the highest reduction potential (Table 3).

**Figure 7:**
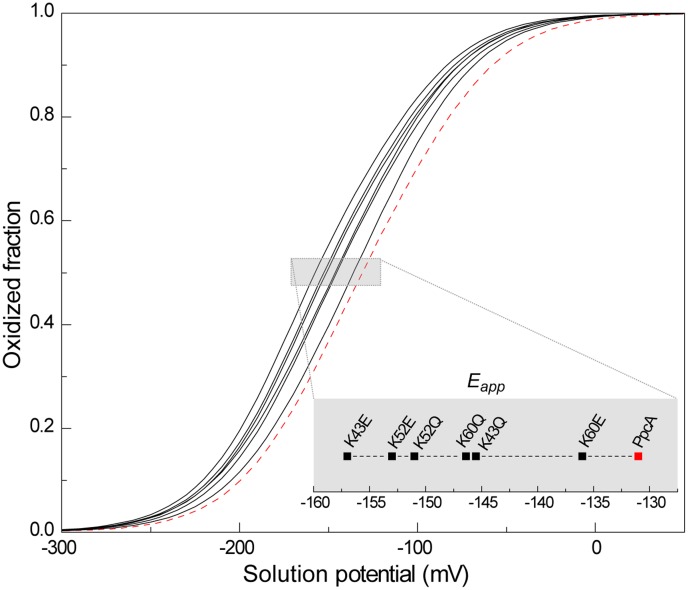
Macroscopic oxidation curves of PpcA mutants (black solid lines) and wild-type (red dashed line) at pH 7.5. The curves were calculated as a function of the solution reduction potential using the parameters listed in Table 2. The midpoint reduction potentials of proteins (*E_app_*) are indicated in the inset.

Finally, the impact of the changes in the heme redox potentials, heme-heme and redox-Bohr interactions on the macroscopic behaviour of K43, K52 and K60 mutants were evaluated. The global oxidation curves of these mutants are depicted in Fig. 7. In all cytochromes, the *E_app_* values (*i.e.* the point at which the oxidized and reduced fractions of each protein are equal) are more negative compared to PpcA indicating that the functional working potential ranges in the mutants are shifted to lower redox potential ranges. The lower *E_app_* values of the mutants are positioned to thermodynamically favour the reduction of downstream redox partners in the *Gs* respiratory chain.

## Conclusions

In the present work we studied the roles of six positively charged lysine residues on the redox properties of the triheme cytochrome PpcA. The selected residues are located in the vicinity of the heme groups and were replaced separately with glutamine and glutamic acid to evaluate the effects of a neutral and negatively charged residue at each position. The results showed that Lys^9^ (near heme IV), Lys^18^ (near heme I) and Lys^22^ (between hemes I and III) have no effect on the redox properties of the heme groups and are probably involved redox partner recognition. However, this hypothesis can only be fully confirmed upon identification of PpcA redox partners. On the other hand, for Lys^43^ and Lys^52^ (both near heme IV) and Lys^60^ (near heme III) the results showed that these residues are crucial in the selection of microstates that allow the protein to establish preferential e^−^/H^+^ transfer pathways and, therefore, in the regulation of the functional mechanism of PpcA. Indeed, the removal of positive charges at positions 43, 52 or 60 altered the redox properties of the hemes and the balance of the global network of cooperativities. The data obtained suggests that the preferred e^−^/H^+^ transfer pathway observed for PpcA is strongly dependent on the reduction potential of heme III relative to the other hemes. In fact, when the reduction potential of heme III is similar to the reduction potentials of the other two hemes, as in the case of K60 mutants, the preferential pathway for electron transfer is disrupted. In contrast, when heme III is clearly the last one to oxidize in native, K43 or K52 mutants, there is a well-established e^−^/H^+^ transfer pathway that involves a single or a two electron transfer coupled to proton transfer. The latter is favored by a larger difference between the reduction potential of heme III and its predecessor in the order of oxidation. Taken together the information obtained in the present and previous studies, we show that this feature is independent from the order of oxidation of the first two hemes and that the functional mechanism of PpcA relies on a fine tuned balance of redox and redox-Bohr interactions to assure a coherent electron transfer pathway coupled to proton transfer that allows the protein to perform e^−^/H^+^ energy transduction. The detailed characterization of the redox properties of PpcA mutants will enable rational design of functional PpcA-based proteins operating at different working potential windows, but, more importantly, preserving the coherent electron transfer pathways that contribute to cellular energy transduction. Having been successful in the design of proteins that work at more negative working potential ranges, with the concomitant increase of the driving force for electron transfer, this work sets the foundations to develop strains carrying mutant cytochromes rationally designed to increase respiratory capabilities of *Geobacter* species.

## Supporting Information

Figure S1 **Alignment of** ***G. sulfurreducens*** **triheme cytochromes PpcA-E amino acid sequences**. The conserved residues are boxed: non-heme attached in black and heme attached in gray, with the corresponding heme number shown below in Roman numerals. For cytochromes PpcB-E, the percentage of sequence identity in relation to PpcA is indicated. The percentage of lysine residues in each protein are shown in parentheses.
(TIF)

Figure S2 **Electronic distribution scheme for a triheme cytochrome with a proton-linked equilibrium showing the 16 possible microstates.** The green and orange circles correspond to the protonated and deprotonated microstates, respectively. Inner circles represent heme groups, which can be either reduced or oxidized and are colored gray or white, respectively. The microstates are grouped, according to the number of oxidized hemes, in four oxidation stages connected by three one-electron redox steps. *P_0H_* and *P_0_* represent the reduced protonated and deprotonated microstates, respectively. *P_ijkH_* and *P_ijk_*, indicate respectively the protonated and deprotonated microstates, where *i*, *j*, and *k* represent the heme(s) that are oxidized in that particular microstate.
(TIF)

Figure S3 **Diagram of a heme c numbered according to the IUPAC-IUB nomenclature** [30] **.**
(TIF)

Figure S4 **Comparison of the observed heme proton chemical shifts of reduced PpcA lysine mutants and those of PpcA at pH 8 and 288K.** The symbols correspond to the glutamine (▵) and the glutamic acid mutants (○). Green, orange, and blue symbols correspond to hemes I, III, and IV, respectively. The rmsd values calculated from the chemical shifts measured for the wild-type and K9, K18 and K22 mutants are 0.01 ppm for all the heme groups, while for the other mutants the rmsd values are: (i) K43Q/E: 0.01; 0.01 and 0.02 ppm, hemes I, III and IV, respectively; (ii) K52Q: 0.01; 0.03 and 0.05 ppm, (iii) K52E: 0.02; 0.05; and 0.08 ppm; (iv) K60Q/E: 0.01; 0.02 and 0.01 ppm. The solid line has a unit slope.
(TIF)

Figure S5 **Expansions of 2D-^1^H EXSY NMR spectra obtained for PpcA and PpcAK mutants at different levels of oxidation (288 K and pH 8).** The 2D-^1^H EXSY NMR spectra of K9, K18 and K22 mutants are similar to those of PpcA and are not represented. Cross-peaks resulting from intermolecular electron transfer between the oxidation stages 1-3 are indicated for the heme methyls 12^1^CH_3_^I^ (green dashed lines), 7^1^CH_3_^III^ (orange dashed lines) and 12^1^CH_3_^IV^ (blue dashed lines). Roman and Arabic numbers indicate the hemes and the oxidation stages, respectively. In order not to overcrowd the figure, the cross-peaks to oxidation stage 0 are not shown. The chemical shifts correspondent to the oxidation stage 0 for each heme methyl are listed in Table S1.
(TIF)

Table S1 **Chemical shifts (ppm) of the heme protons of mutants in the reduced state at pH 8 and 288K.** For comparison chemical shift values obtained for PpcA [10] are indicated in parenthesis.
(DOCX)
