# Supplementary figures and images for: Dissecting the Functional Role of Key Residues in Triheme Cytochrome PpcA: A Path to Rational Design of G. sulfurreducens Strains with Enhanced Electron Transfer Capabilities
Source: PLoS One. 2014 Aug 25;9(8):e105566. doi: 10.1371/journal.pone.0105566 (PMC4143306; doi:10.1371/journal.pone.0105566)

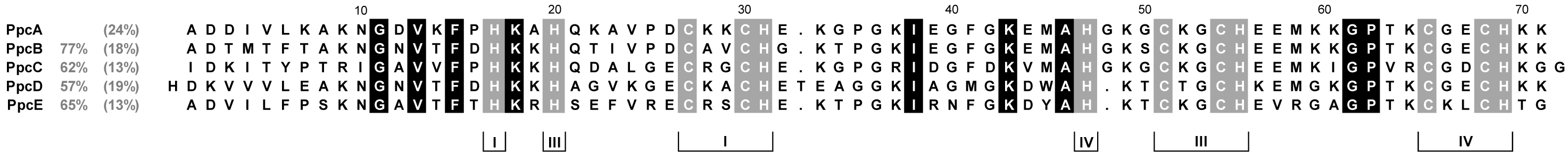

Supplement: Figure S1 — Alignment of G. sulfurreducens triheme cytochromes PpcA-E amino acid sequences. The conserved residues are boxed: non-heme attached in black and heme attached in gray, with the corresponding heme number shown below in Roman numerals. For cytochromes PpcB-E, the percentage of sequence identity in relation to PpcA is indicated. The percentage of lysine residues in each protein are shown in parentheses. (TIF) [file pone.0105566.s001.tif]

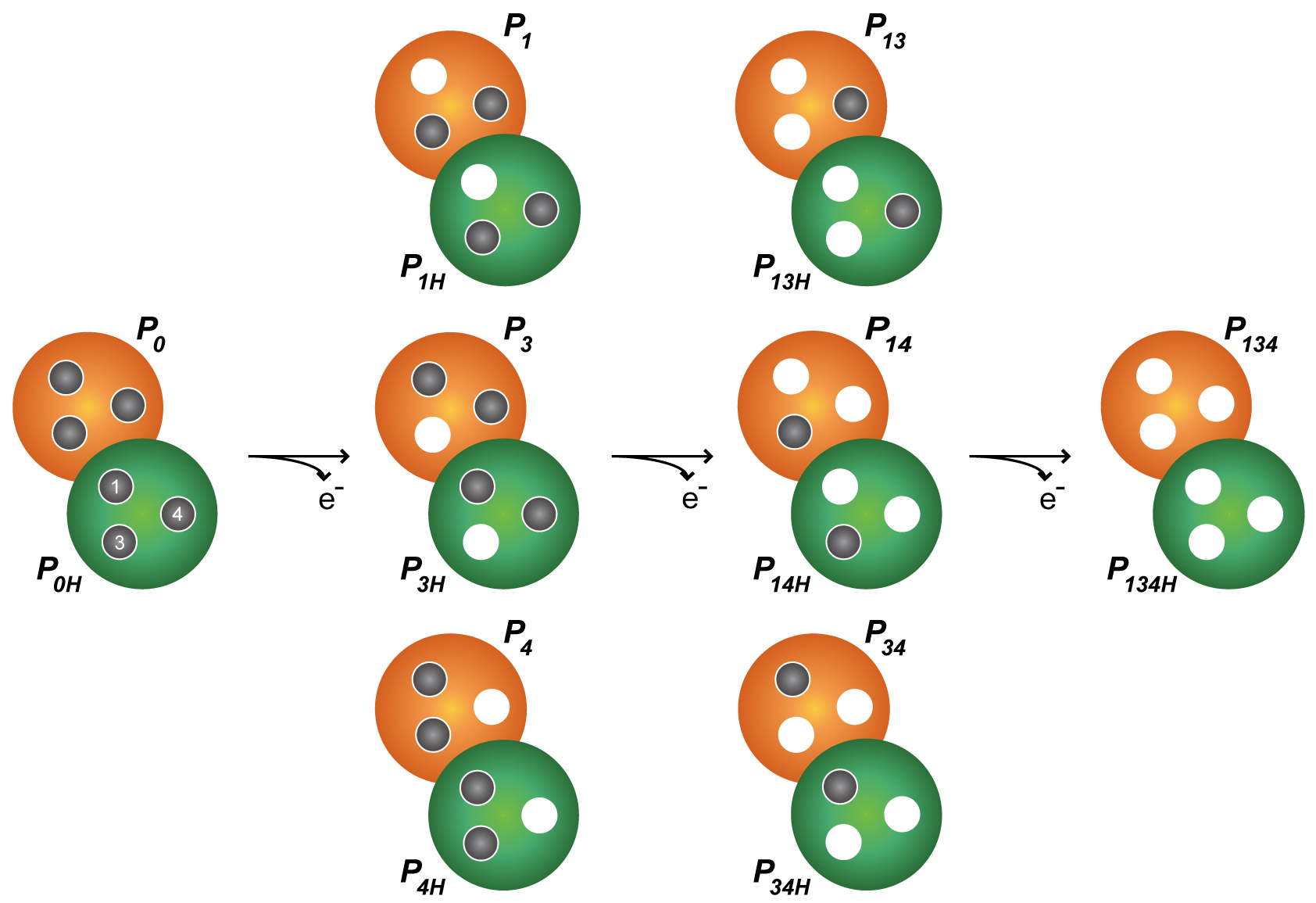

Supplement: Figure S2 — Electronic distribution scheme for a triheme cytochrome with a proton-linked equilibrium showing the 16 possible microstates. The green and orange circles correspond to the protonated and deprotonated microstates, respectively. Inner circles represent heme groups, which can be either reduced or oxidized and are colored gray or white, respectively. The microstates are grouped, according to the number of oxidized hemes, in four oxidation stages connected by three one-electron redox steps. P0H and P0 represent the reduced protonated and deprotonated microstates, respectively. PijkH and Pijk, indicate respectively the protonated and deprotonated microstates, where i, j, and k represent the heme(s) that are oxidized in that particular microstate. (TIF) [file pone.0105566.s002.tif]

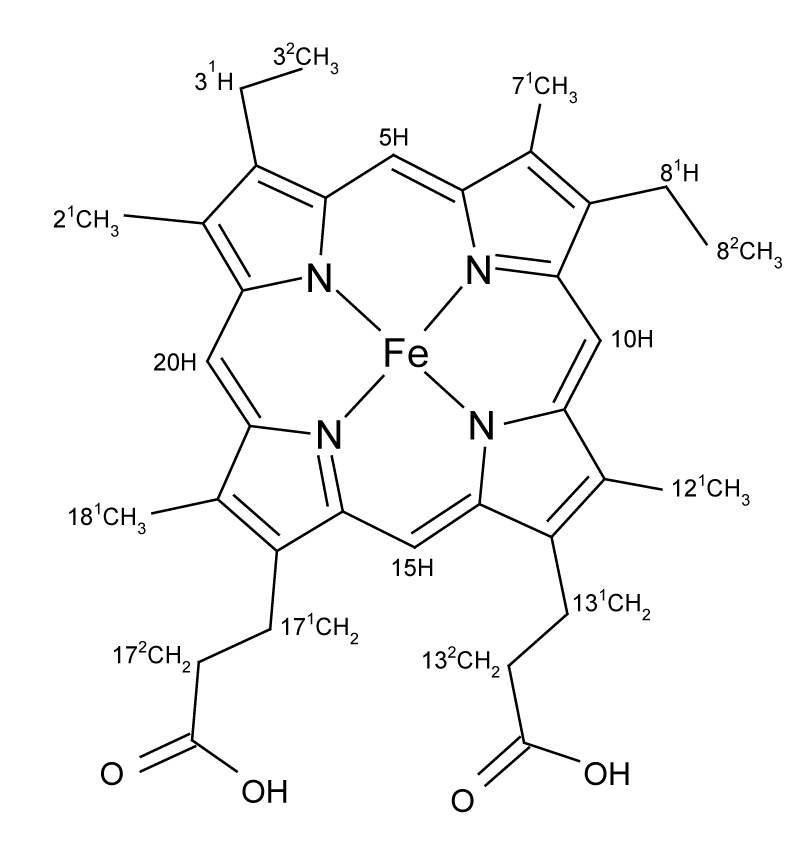

Supplement: Figure S3 — Diagram of a heme c numbered according to the IUPAC-IUB nomenclature [30] . (TIF) [file pone.0105566.s003.tif]

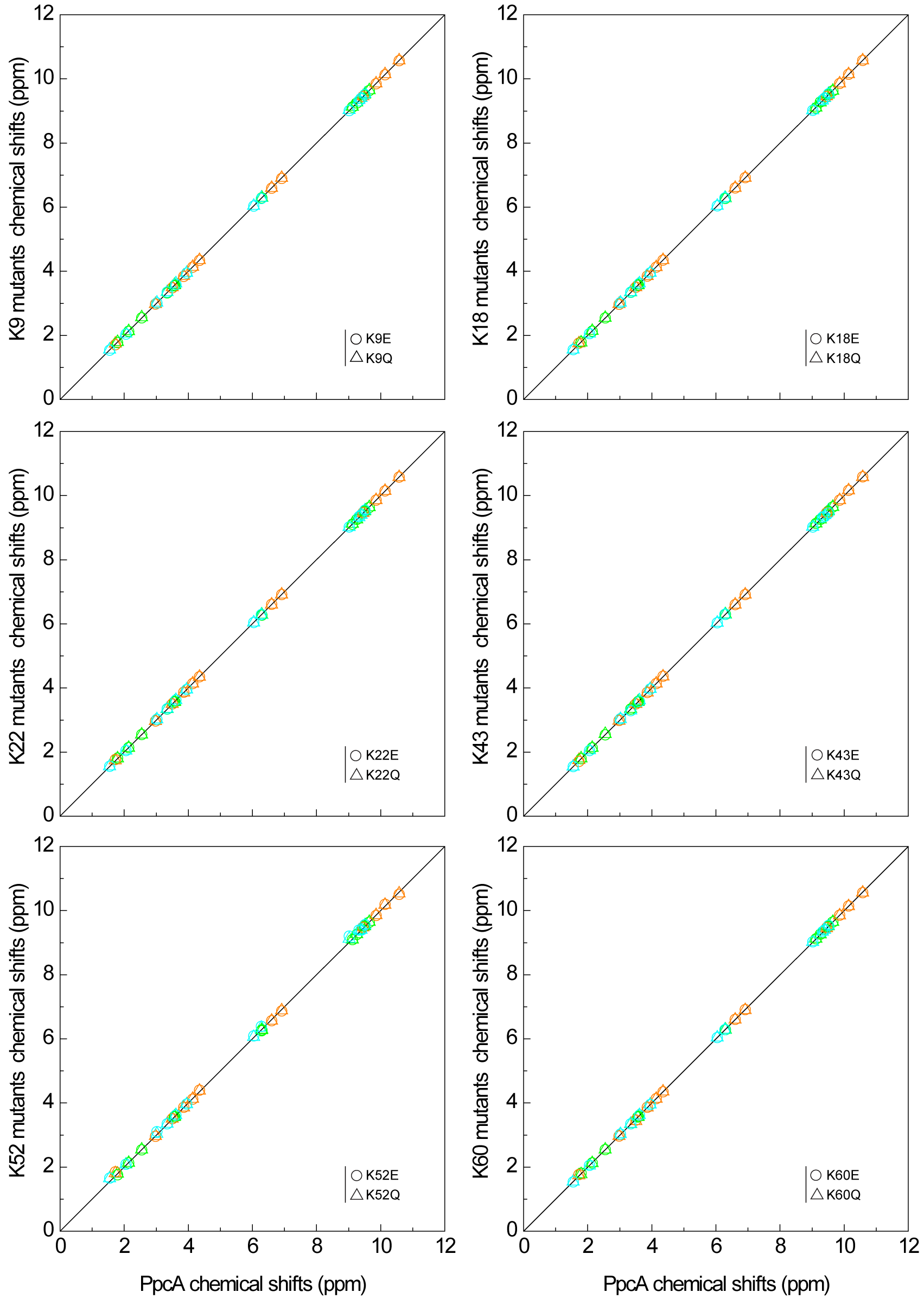

Supplement: Figure S4 — Comparison of the observed heme proton chemical shifts of reduced PpcA lysine mutants and those of PpcA at pH 8 and 288K. The symbols correspond to the glutamine (▵) and the glutamic acid mutants (○). Green, orange, and blue symbols correspond to hemes I, III, and IV, respectively. The rmsd values calculated from the chemical shifts measured for the wild-type and K9, K18 and K22 mutants are 0.01 ppm for all the heme groups, while for the other mutants the rmsd values are: (i) K43Q/E: 0.01; 0.01 and 0.02 ppm, hemes I, III and IV, respectively; (ii) K52Q: 0.01; 0.03 and 0.05 ppm, (iii) K52E: 0.02; 0.05; and 0.08 ppm; (iv) K60Q/E: 0.01; 0.02 and 0.01 ppm. The solid line has a unit slope. (TIF) [file pone.0105566.s004.tif]

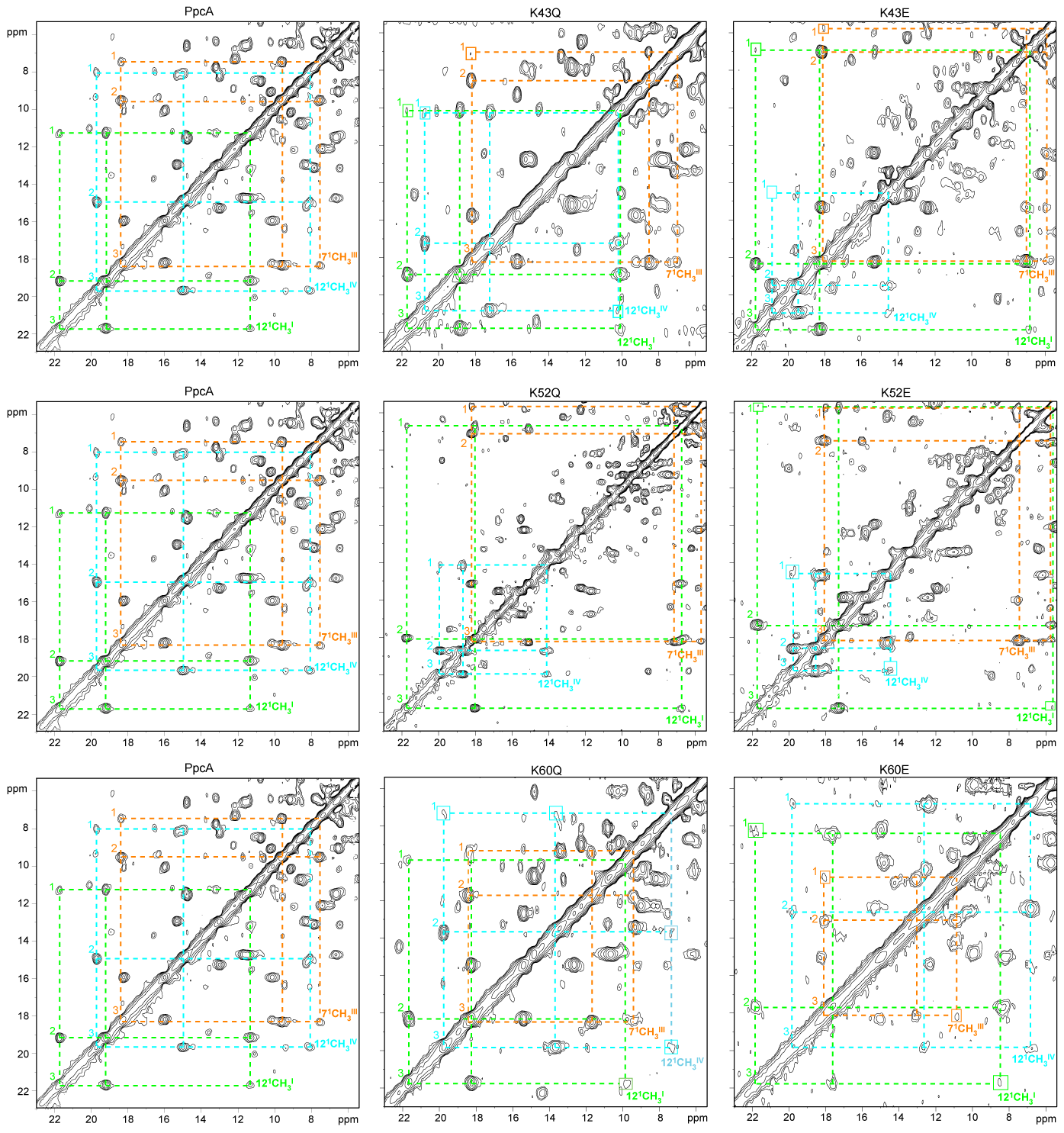

Supplement: Figure S5 — Expansions of 2D-1H EXSY NMR spectra obtained for PpcA and PpcAK mutants at different levels of oxidation (288 K and pH 8). The 2D-1H EXSY NMR spectra of K9, K18 and K22 mutants are similar to those of PpcA and are not represented. Cross-peaks resulting from intermolecular electron transfer between the oxidation stages 1-3 are indicated for the heme methyls 121CH3 I (green dashed lines), 71CH3 III (orange dashed lines) and 121CH3 IV (blue dashed lines). Roman and Arabic numbers indicate the hemes and the oxidation stages, respectively. In order not to overcrowd the figure, the cross-peaks to oxidation stage 0 are not shown. The chemical shifts correspondent to the oxidation stage 0 for each heme methyl are listed in Table S1. (TIF) [file pone.0105566.s005.tif]
