# Supplementary material for: Dissecting the Functional Role of Key Residues in Triheme Cytochrome PpcA: A Path to Rational Design of G. sulfurreducens Strains with Enhanced Electron Transfer Capabilities
Source: PLoS One. 2014 Aug 25;9(8):e105566. doi: 10.1371/journal.pone.0105566 (PMC4143306; doi:10.1371/journal.pone.0105566)
Supplement: Table S1 — Chemical shifts (ppm) of the heme protons of mutants in the reduced state at pH 8 and 288K. For comparison chemical shift values obtained for PpcA [10] are indicated in parenthesis. (DOCX) [file pone.0105566.s006.docx]

Table S1 - Chemical shifts (ppm) of the heme protons of mutants in the reduced state at pH 8 and 288K. For comparison chemical shift values obtained for PpcA are indicated in parenthesis.

| **Heme substituent** | **Mutant** | **Heme I** | **Heme III** | **Heme IV** |
| --- | --- | --- | --- | --- |
| 5H | K9Q  K9E  K18Q  K18E  K22Q  K22E  K43Q  K43E  K52Q  K52E  K60Q  K60E | 9.65 (9.65)  9.64  9.63  9.63  9.64  9.63  9.65  9.65  9.65  9.64  9.65  9.65 | 10.58 (10.58)  10.57  10.58  10.58  10.58  10.58  10.58  10.59  10.54  10.51  10.56  10.56 | 9.03 (9.02)  9.01  9.02  9.02  9.02  9.02  9.03  9.02  9.12  9.21  9.02  9.03 |
| 10H | K9Q  K9E  K18Q  K18E  K22Q  K22E  K43Q  K43E  K52Q  K52E  K60Q  K60E | 9.13 (9.12)  9.12  9.11  9.10  9.12  9.11  9.13  9.13  9.10  9.10  9.12  9.12 | 9.86 (9.86)  9.85  9.86  9.86  9.86  9.86  9.86  9.86  9.86  9.85  9.85  9.86 | 9.34 (9.33)  9.32  9.33  9.34  9.33  9.34  9.35  9.33  9.38  9.39  9.34  9.34 |
| 15H | K9Q  K9E  K18Q  K18E  K22Q  K22E  K43Q  K43E  K52Q  K52E  K60Q  K60E | 9.27 (9.26)  9.25  9.27  9.28  9.26  9.26  9.26  9.26  9.27  9.26  9.26  9.27 | 9.46 (9.45)  9.45  9.46  9.45  9.46  9.47  9.46  9.46  9.48  9.48  9.45  9.47 | 9.54 (9.51)  9.51  9.54  9.54  9.54  9.55  9.54  9.45  9.55  9.56  9.53  9.54 |
| 20H | K9Q  K9E  K18Q  K18E  K22Q  K22E  K43Q  K43E  K52Q  K52E  K60Q  K60E | 9.51 (9.50)  9.50  9.51  9.52  9.51  9.51  9.51  9.51  9.51  9.51  9.51  9.51 | 10.15 (10.14)  10.13  10.15  10.15  10.15  10.15  10.16  10.16  10.18  10.20  10.14  10.15 | 9.40 (9.39)  9.39  9.40  9.40  9.40  9.40  9.39  9.35  9.40  9.39  9.40  9.40 |

*(continued on next page)*

Table S1*(continued)*

| **Heme substituent** | **Mutant** | **Heme I** | **Heme III** | **Heme IV** |
| --- | --- | --- | --- | --- |
| 2^1^CH_3_ | K9Q  K9E  K18Q  K18E  K22Q  K22E  K43Q  K43E  K52Q  K52E  K60Q  K60E | 3.56 (3.56)  3.55  3.56  3.56  3.56  3.56  3.56  3.56  3.56  3.57  3.56  3.56 | 4.35 (4.35)  4.34  4.35  4.35  4.35  4.35  4.36  4.37  4.38  4.40  4.36  4.35 | 3.62 (3.61)  3.60  3.62  3.62  3.62  3.62  3.61  3.58  3.62  3.62  3.62  3.62 |
| 7^1^CH_3_ | K9Q  K9E  K18Q  K18E  K22Q  K22E  K43Q  K43E  K52Q  K52E  K60Q  K60E | 3.58 (3.58)  3.56  3.57  3.57  3.57  3.57  3.58  3.58  3.58  3.57  3.57  3.58 | 4.14 (4.14)  4.13  4.13  4.13  4.14  4.14  4.14  4.14  4.13  4.12  4.13  4.14 | 3.02 (3.02)  3.01  3.02  3.02  3.02  3.02  3.03  3.04  3.04  3.11  3.03  3.03 |
| 12^1^CH_3_ | K9Q  K9E  K18Q  K18E  K22Q  K22E  K43Q  K43E  K52Q  K52E  K60Q  K60E | 2.56 (2.55)  2.55  2.55  2.55  2.55  2.55  2.56  2.54  2.55  2.54  2.55  2.55 | 3.50 (3.50)  3.49  3.50  3.50  3.50  3.50  3.50  3.50  3.51  3.51  3.44  3.47 | 3.95 (3.95)  3.94  3.95  3.95  3.95  3.95  3.97  3.97  3.96  3.97  3.95  3.95 |
| 18^1^CH_3_ | K9Q  K9E  K18Q  K18E  K22Q  K22E  K43Q  K43E  K52Q  K52E  K60Q  K60E | 3.35 (3.34)  3.33  3.34  3.34  3.34  3.34  3.35  3.34  3.35  3.35  3.34  3.35 | 3.86 (3.86)  3.84  3.85  3.85  3.86  3.87  3.86  3.86  3.86  3.87  3.86  3.87 | 3.35 (3.34)  3.35  3.35  3.35  3.35  3.35  3.30  3.29  3.35  3.35  3.35  3.35 |

*(continued on next page)*

Table S1*(continued)*

| **Heme substituent** | **Mutant** | **Heme I** | **Heme III** | **Heme IV** |
| --- | --- | --- | --- | --- |
| 3^1^H | K9Q  K9E  K18Q  K18E  K22Q  K22E  K43Q  K43E  K52Q  K52E  K60Q  K60E | 6.30 (6.30)  6.29  6.28  6.28  6.28  6.27  6.30  6.30  6.29  6.28  6.29  6.29 | 6.91 (6.91)  6.90  6.91  6.92  6.91  6.91  6.91  6.91  6.89  6.87  6.90  6.91 | 6.04 (6.04)  6.03  6.04  6.04  6.04  6.04  6.03  6.03  6.06  6.09  6.04  6.04 |
| 8^1^H | K9Q  K9E  K18Q  K18E  K22Q  K22E  K43Q  K43E  K52Q  K52E  K60Q  K60E | 6.29 (6.29)  6.29  6.28  6.27  6.29  6.29  6.29  6.30  6.27  6.25  6.28  6.29 | 6.60 (6.60)  6.59  6.60  6.60  6.60  6.61  6.60  6.60  6.57  6.57  6.60  6.61 | 6.29 (6.28)  6.27  6.29  6.29  6.28  6.29  6.29  6.30  6.35  6.39  6.29  6.29 |
| 3^2^CH_3_ | K9Q  K9E  K18Q  K18E  K22Q  K22E  K43Q  K43E  K52Q  K52E  K60Q  K60E | 2.13 (2.14)  2.12  2.14  2.13  2.13  2.14  2.13  2.13  2.13  2.13  2.12  2.13 | 1.74 (1.73)  1.71  1.75  1.76  1.74  1.77  1.75  1.71  1.81  1.85  1.74  1.77 | 2.06 (2.06)  2.04  2.06  2.06  2.05  2.05  2.06  2.05  2.08  2.09  2.05  2.06 |
| 8^2^CH_3_ | K9Q  K9E  K18Q  K18E  K22Q  K22E  K43Q  K43E  K52Q  K52E  K60Q  K60E | 1.79 (1.79)  1.78  1.78  1.79  1.80  1.81  1.80  1.79  1.79  1.76  1.78  1.81 | 2.98 (2.98)  2.98  2.98  2.97  2.97  2.97  2.98  2.99  2.97  2.96  2.97  2.97 | 1.56 (1.55)  1.54  1.56  1.56  1.56  1.56  1.55  1.55  1.65  1.66  1.55  1.54 |
